# Comparison of clinical outcomes of venous thromboembolic disease between outpatient and inpatient management

**DOI:** 10.47487/apcyccv.v4i4.334

**Published:** 2024-03-19

**Authors:** Felipe Aníbal Gregalio, Camila Juana, Gian Manattini Palmili, Bernardo Julio Martínez, Ignacio Martin Bluro, Fernando Javier Vázquez, María Florencia Grande Ratti

**Affiliations:** 1 Servicio de Clínica Médica, Hospital Italiano de Buenos Aires, Buenos Aires, Argentina. Servicio de Clínica Médica Hospital Italiano de Buenos Aires Buenos Aires Argentina; 2 Instituto Universitario Hospital Italiano de Buenos Aires, Buenos Aires, Argentina Instituto Universitario del Hospital Italiano Instituto Universitario Hospital Italiano de Buenos Aires Buenos Aires Argentina; 3 Central de Emergencias de Adultos, Hospital Italiano de Buenos Aires, Buenos Aires, Argentina. Central de Emergencias de Adultos Hospital Italiano de Buenos Aires Buenos Aires Argentina; 4 Servicio de Cardiología, Hospital Italiano de Buenos Aires, Buenos Aires, Argentina. Servicio de Cardiología Hospital Italiano de Buenos Aires Buenos Aires Argentina; 5 CONICET-IMTIB, Instituto Universitario Hospital Italiano de Buenos Aires, Buenos Aires, Argentina. Instituto Universitario del Hospital Italiano CONICET-IMTIB, Instituto Universitario Hospital Italiano de Buenos Aires Buenos Aires Argentina; 6 Área de Investigación en Medicina Interna, Hospital Italiano de Buenos Aires, Buenos Aires, Argentina. Área de Investigación en Medicina Interna Hospital Italiano de Buenos Aires Buenos Aires Argentina; 7 CONICET-HIBA, Instituto Universitario Hospital Italiano de Buenos Aires, Buenos Aires, Argentina. Instituto Universitario del Hospital Italiano CONICET-HIBA, Instituto Universitario Hospital Italiano de Buenos Aires Buenos Aires Argentina

**Keywords:** Emergency Service, Hospital, Venous thromboembolism, Pulmonary embolism, Argentina, Ambulatory care

## Abstract

**Objectives.:**

To compare the occurrence of death, bleeding, and recurrence according to inpatient or outpatient management of venous thromboembolic disease (VTE).

**Materials and methods:**

. Retrospective cohort that included a consecutive sampling of VTE consultations between 2016 and 2019 diagnosed in the Emergency Center of a private hospital in Argentina.

**Results.:**

There were 1202 cases, 908 with isolated deep vein thrombosis (DVT), 205 with isolated pulmonary embolism (PE), and 89 cases of combined DVT - PE. 66% were women, with a median age of 77 years; 72% of cases were managed on an outpatient basis (n= 862). Comorbidities associated with hospitalization were obesity (p=0.03), chronic obstructive pulmonary disease (COPD) (p=0.01), heart failure (CHF) (p=0.01), chronic renal failure (CKD) (p=0.01), and cancer (p=0.01). At 90 days, the cumulative incidence of bleeding was 2.6% in inpatient compared to 2.9% in outpatient management (p=0.81); recurrence was 0% versus 0.9% (p=0.07), and mortality was 42.9% versus 18.9%, respectively (p=0.01). The HR for 90-day mortality in hospitalized patients adjusted for confounders (sex, age, type of VTE, obesity, CKD, CHF, COPD, and cancer) was 1.99 (95% CI 1.49-2.64; p=0.01).

**Conclusions.:**

In this elderly, and predominantly female Argentine population, the 90-day mortality in patients hospitalized for VTE was higher than mortality in patients with outpatient management, without differences in recurrence or major bleeding.

## Introduction

The diagnosis and therapeutic approach of venous thromboembolic disease (VTE) has changed drastically due to the emergence of acute out-of-hospital management, especially with the introduction of new oral anticoagulants ^1^. Whereas, historically, all patients were admitted and hospitalized for anticoagulation treatment and follow-up; recent literature suggests that outpatient treatment is safe, feasible and effective, with similar rates of recurrence and all-cause mortality ^2^.

Despite the clear potential benefits of outpatient care (reducing healthcare costs and not requiring patients to change their environment), most people suffering from pulmonary embolism (PE) continue to be hospitalized due to fear of possible severe adverse events, thus pursuing the goal of early discharge. An American study that included cases of VTE between 2011 and 2018 showed that outpatient treatment was used for 57% of deep vein thrombosis (DVT), but only 18% of PE ^3^. Consistently, a study from Argentina found that outpatient management of overall VTE was 72%, but when stratified by subtype, 89% was for DVT and only 19% for PE ^4^.

The overall rate of early complications (i.e. between 1 and 3 months of follow-up) was <2% for recurrent thromboembolic events and/or major bleeding, and <3% for all-cause mortality, with no evidence in favour of either strategy ^5^. However, there is uncertainty about severe complications (such as recurrence, bleeding and/or death) according to initial approach: outpatient versus inpatient. Therefore, the present study aimed to compare the occurrence of death (primary outcome), recurrence and bleeding (secondary outcomes, with death as a competing event) at 90 days after the diagnosis of VTE.

## Materials y methods

### Design and population

A retrospective cohort that included consecutive sampling of all VTE diagnosed at the Adult Emergency Centre (AEC) of the Hospital Italiano in Buenos Aires, between January 1, 2016 and December 31, 2019, corresponding to members of the institutional prepaid health insurance (restriction explained by the fact that it is a closed cohort with reliable secondary data on follow-up).

Patients were followed from the time of VTE diagnosis in the emergency department until the occurrence of death (primary outcome), recurrence and/or bleeding (secondary outcomes, with death as a competing event), loss of follow-up (e.g. disaffiliation from prepaid health insurance, discharge due to debt) or administrative censorship (90 days). Sampling was consecutive with a fixed number of subjects during the study period, without the need for a sample size calculation.

### Procedures

All patient health information is stored in a single clinical data repository (CDR) fed by the hospital’s electronic health record (EHR), evaluated and accredited by the Healthcare Information and Management Systems Society as Level 7+. This comprehensive CDR stores clinical documents for each patient, from different sources such as test results, images, clinical notes, outpatient visits, emergency room visits, hospital care, among others. We use these high-quality secondary databases for data collection.

Potential cases of VTE were identified through the medical record at the epicrisis closure in the electronic health record, using the Pan American Health Organization’s International Classification of Diseases version 10 (known as ICD-10). Subsequently, there was a manual review by experts for validation. VTE was defined as acute symptomatic DVT and/or acute symptomatic PE.

Cases were classified as hospitalization or outpatient management (exposure variable), based on emergency discharge status; that is, based on initial approach at the time of diagnosis. Home hospitalization is a care model that provides hospital-level services at the patient’s home as an alternative to hospitalization. These programs typically involve a multidisciplinary team of healthcare professionals, including physicians, nurses and other healthcare providers, working together to provide comprehensive and coordinated care to patients in their homes. Home hospitalization was considered as outpatient management, as was home discharge.

### Study variables

The most important study variables were: VTE, defined as a diagnosis of DVT (confirmed by Doppler ultrasound or upper and lower limb angiography) and/or PE (confirmed by angiotomography, angiogram and intermediate or high probability VQ scintigraphy). Recurrence as the occurrence of a new VTE event within 72 hours of the initial diagnosis. Major bleeding as any event that resulted in a hemoglobin decrease of ≥2 g/dL and/or requiring transfusion of at least two units of red blood cells and/ or intracranial, retroperitoneal, intraocular bleeding or any event requiring hospitalization as recorded in electronic health record during follow-up. Mortality as death from any cause within 90 days after the index episode of VTE, which may occur in-hospital or out-of-hospital (collected from the register, where the reason for disaffiliation from the prepaid health insurance is recorded).

### Data analysis

Statistical analysis was performed with STATA 18.0 (Stata Corporation, College Station, TX). Descriptive statistics were used, and quantitative variable data were presented as mean and standard deviation (SD) or median and interquartile range (IQR), depending on the distribution. Categorical variables were reported as absolute and relative frequencies.

Additionally, analytical statistics were used to compare groups according to management (outpatient vs. inpatient). Chi-square or Fisher’s exact test was used for categorical variables, and Mann-Whitney test or Student’s t test for continuous variables. Statistical significance was considered for p-values <0.05.

Time to event was used to report cumulative incidences at 90 days, and Cox regression was employed, reporting Hazard Ratios (HR) with their respective 95% confidence intervals (CI), crude (HR) and adjusted (aHR) for potential confounders. 90-day survival in both groups was analyzed through Kaplan-Meier curves.

### Ethical considerations

This project was developed in compliance with ethical principles consistent in accordance with national and international regulatory standards for human health research. The protocol was approved by the Institutional Ethics Committee (CEPI#5659). As this was an observational and retrospective study, the signed informed consent of the participating subjects was not required.

## Results

During the study period, there were 1202 cases of VTE: 908 DVT alone (75%), 205 PE alone (17%) and 89 with both DVT and PE (8%). The majority were female patients (65.8%) and elderly with median age of 77 ± 13 years, 81% were over 65 years old, and 42% were 80 years or older. The most common risk factors were obesity (44%) and cancer (27%).

**Figure 1:**
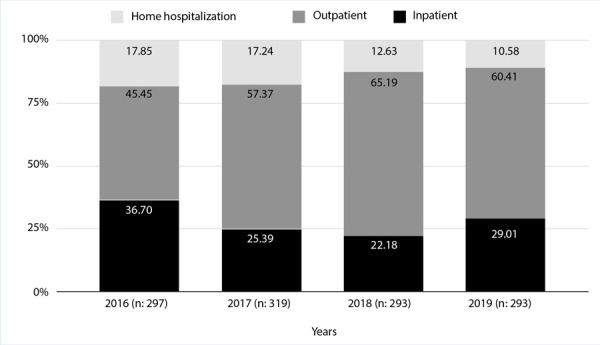
Evolution throughout the years 2016-2019 of VTE management stratified by groups (home hospitalization, outpatient, and inpatient).

As observed in Figure 1, there was a reduction in hospitalizations over these years (from 36% in 2016 to 29% in 2019; p=0.046), not at the expense of home hospitalization, but as a result of an increase in outpatient management (from 45% in 2016 to 60% in 2019; p=0.001).

According to initial management, 72% of cases were outpatient and only 28% required hospitalization (Table 1). Comorbidities associated with hospitalization of one case were obesity (p=0.03), chronic obstructive pulmonary disease (COPD) (p =0.01), heart failure (HF) (p=0.01), chronic kidney disease (CKD) (p=0.01), and cancer (p=0.01). Only 25 patients received direct oral anticoagulants (DOACs) as first-line drug in emergencies at the time of diagnosis.

**Table 1:** Baseline characteristics of the population (n=1202).

	Outpatient n=862 % (n)	Inpatient n=340 % (n)	p value
Type of VTE			0.001
DVT alone	94.08% (811)	28.53% (97)	
PE alone	4.52% (39)	48.82% (166)	
DVT-PE	1.39% (12)	22.65% (77)	
Baseline epidemiological characteristics			
Age, in years **	77 (68-84)	78 (69-85)	0.056
Female sex	64.2% (553)	70% (238)	0.054
Obesity	42.1% (363)	48.8% (166)	0.035
Hypertension	64.2% (553)	70% (238)	0.054
Stroke	6.3% (54)	7.1% (24)	0.615
COPD	7.1% (61)	12.7% (43)	0.002
Pulmonary fibrosis	0.2% (2)	1.5% (5)	0.011
Chronic kidney disease	4.6% (40)	8.5% (29)	0.009
Heart failure	5.3% (46)	10.9% (37)	0.001
Trasplant	0.7% (6)	1.8% (6)	0.093
Categorical Charlson			
Mild	71.6% (617)	52.7% (179)	0.001
Moderate	15.6% (134)	25.6% (87)	
Severe	12.9% (111)	21.8% (74)	
VTE risk factors			
Cancer	23.8% (205)	34.1% (116)	0.001
Prior VTE	2.1% (18)	2.4% (8)	0.776
Thrombophilia	0.4% (3)	0.6% (2)	0.560
Bleeding risk variables			
Prior major bleeding	4.2% (36)	5.6% (19)	0.291
Aspirin use	46.4% (400)	47.7% (162)	0.697
Clopidogrel use	7.3% (63)	8.5% (29)	0.473
Cilostazol use	3.9% (34)	2.7% (9)	0.275
Treatment-related variables					
No treatment ***	7.1% (61)	2.9% (10)	0.001
Enoxaparin	82.7% (713)	87.7% (298)	0.034
DOACs	2.9% (25)	0% (0)	0.001
Acenocoumarol	1.4% (12)	0% (0)	0.028
Heparin	0% (0)	1.8% (6)	0.001
Inferior vena cava filter or Thrombolytic	0.1% (1)	5.6% (19)	0.001
More than one drug	5.8% (50)	2.1% (7)	0.006

** Median, interquartile range (IQR)

*** No treatment due to death in the emergency room, contraindication, palliative care, or end of life.

DOACs: Direct Oral Anticoagulants; COPD: Chronic Obstructive Pulmonary Disease; VTE: Venous Thromboembolic Disease; PE: Pulmonary Embolism; DVT: Deep Vein Thrombosis

**Figure 2:**
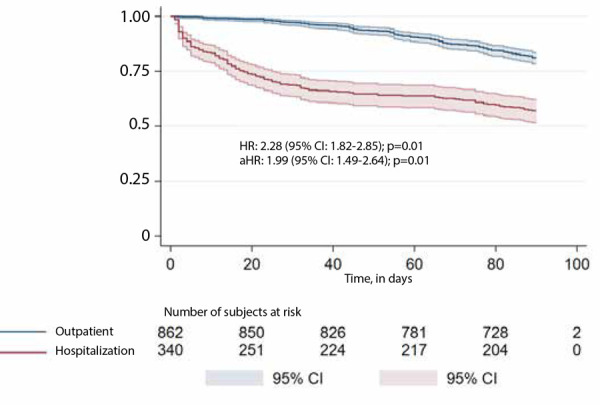
90-day survival according to initial management strategy

Table 2 shows the time-to-event analysis of the different outcomes of interest. When it comes to all-cause mortality, the cumulative incidence at 90 days was 42.9% in hospitalized patients versus 18.9% in outpatient patients (HR: 2.28; 95% CI: 1.82-2.85; p=0.001). After adjusting for clinically relevant and statistically significant covariates (sex, age, type of VTE, obesity, CKD, HF, and cancer), aHR was 1.99 (95% CI: 1.49-2.64; p=0.001). As shown in Figure 2, most deaths in the hospitalized group were early (within a 72-hour time window), with a median time to the event of 1 day; the most common causes of death: sepsis (intercurrent), cancer (underlying pathology) or PE.

**Table 2:** Analysis of time to event (cumulative incidences at 90 days).

	VTE (n=1202)	Outpatient (n=862)	Inpatient (n=340)	p value
Death at 90 days	25.7% (309)	18.9% (163)	42.9% (146)	0.001
Bleeding at 90 days	2.8% (34)	2.9% (25)	2.7% (9)	0.812
Recurrence at 90 days	0.7% (8)	0.9% (8)	0% (0)	0.075
Hospitalization at 90 days	-	15.1% (130)	-	N/A

VTE: venous thromboembolism

N/A: not aplicable

HR: crude Hazard Ratio

a HR: Hazard Ratio adjusted for clinically relevant and statistically significant covariates: sex, age, type of VTE, obesity, chronic kidney disease, heart failure, chronic obstructive pulmonary disease, and cancer.

The 90-day recurrence had a cumulative incidence of 0% in hospitalized versus 1% in outpatient (p=0.075); while the 90-day bleeding was 2.6% in inpatient versus 2.9% in outpatient (p=0.812). Among the 862 outpatient cases, the 90-day hospitalization rate was 15%.

Restricting the analysis to PTE cases, the overall 30-day mortality was 37.75% with no differences between inpatient and outpatient (31.37% and 39.09%; p=0.301). In DVT cases, the overall 30-day mortality rate was 22.9%, higher in inpatient compared to outpatient cases (46.6% and 17.9%, respectively; p=0.001).

Table 3 shows the factors associated with hospitalization in PE cases. Associated factors were: higher PESI score (56% were classified as IV or V), and the higher complementary studies such as serological troponin dosing (Odds Ratio [OR]: 1.95; 95% CI: 0.90-4.21; p=0.088) and BNP (OR: 2.63; 95% CI: 1.18-5.81; p=0.017). Consistently, inpatients had higher troponin and BNP values compared to outpatients, with median values of 24.2 ng/L vs. 11.4 ng/ L (p=0.001) and 455.1 pg/mL vs. 141.2 pg/mL (p=0.001), respectively.

**Table 3:** Factors associated with hospitalization in pulmonary embolism cases (n=294).

	Total (n=294)	Outpatient (n=51)	Inpatient (n=243)	p value
Age in years*	74 ± 12.4	72 ± 10.6	75 ± 12.6	0.202
Female sex	69.0% (203)	56.9% (29)	71.6% (174)	0.038
Obesity	48.6% (143)	33.3% (17)	51.9% (126)	0.016
Cancer	36.4% (294)	39.2% (20)	35.8% (87)	0.645
Chronic kidney disease	5.1% (15)	1.9% (1)	5.8% (14)	0.262
Prior major bleeding	2.7% (8)	1.9% (1)	2.9% (7)	0.714
Prior VTE	2.1% (6)	0% (0)	2.5% (6)	0.257
Thrombophilia	0.3% (1)	0% (0)	0.4% (1)	0.646
PESI score				
I	5.4% (16)	7.8% (4)	1.2% (3)	
II	24.8% (73)	35.3% (18)	4.9% (12)	
III	28.2% (83)	17.7% (9)	22.6% (55)	0.142
IV	25.2% (74)	23.5% (12)	30.5% (74)	
V	14.3% (45)	9.8% (5)	25.5% (62)	
No data	2.1% (6)	5.9% (3)	15.2% (37)	
Troponin dosage	86.1% (253)	78.4% (40)	87.7% (213)	0.001
Troponin value, ng/L**	19.7 (11.9-45.1)	11.4 (8.15-16.7)	24.2 (13.4-62.9)	0.001
BNP dosage (%, n)	88.4% (260)	78.4% (40)	90.5% (220)	0.001
BNP value, pgrs**	374 (137-1331)	141 (58-272)	455 (159-1777)	0.001
Echocardiogram	32.7% (96)	47.1% (24)	29.6% (72)	0.016

*Mean and standard deviation

** Median, interquartile range (IQR)

BNP: B-type natriuretic peptide; VTE: Venous thromboembolic; ng/L: nanograms per liter; PESI: Pulmonary Embolism Severity Index; pgrs: picogram

PE: Pulmonary Embolism

As shown in Table 4, factors associated with hospitalization in cases of DVT cases included a history of CKD (OR: 2.75; 95% CI: 1.57-4.82; p=0.001) and prior major bleeding (OR: 1.97; 95% CI: 1.03-3.44; p=0.039).

**Table 4:** Factors associated with hospitalization in DVT cases (n=997).

	Total (n=997)	Outpatient (n=823)	Inpatient (n=174)	p value
Age in years*	74 ± 13.8	73.8 ± 13.9	75.9 ± 13.8	0.070
Female sex	64.9% (647)	64.5% (531)	66.7% (116)	0.590
Obesity	42.7% (426)	42.4% (349)	44.3% (77)	0.655
Cancer	23.9% (239)	22.9% (188)	29.3% (51)	0.069
Chronic kidney disease	6.0% (60)	4.7% (39)	12.1% (21)	0.001
Prior major bleeding	4.9% (49)	4.3% (35)	8.1% (14)	0.035
Prior VTE	2.1% (21)	2.2% (18)	1.7% (3)	0.699
Thrombophilia	0.5% (5)	0.4% (3)	1.2% (2)	0.183

*Mean and standard deviation

VTE: venous thromboembolic; DVT: deep vein thrombosis

## Discussion

The main findings of the study were (a) hospitalization by VTE was associated with more severe cases and patients with multiple comorbidities; (b) at 90 days, mortality was higher in hospitalized patients, but with no differences in recurrence or bleeding, and (c) 15% of patients initially managed on an outpatient basis were hospitalized during follow-up.

Firstly, it is worth mentioning that this is a particularly elderly population (median age of 77 years), probably explained by the fact that the prepaid health insurance in question is one of the few national health insurance companies that actively accepts the admission of older adults and, therefore, 35% of its members are over 60 years of age, which facilitates clinical research in this subpopulation usually excluded from clinical trials (RCTs) ^6^^,^^7^. In this regard, the geriatrics and home medicine sections were pioneers at the local level, currently having extensive experience in the management, achieved through the implementation of a personalized treatment plan, coordinated through the transition care process and co-management ^8^. As evidenced in a local publication, 59,056 people out of a portfolio of 150,725 active partners were >65 years of age, representing 39% of the population in 2019 ^9^. Population ageing is not only an epidemiological phenomenon with implications for public health, social services and the sustainability of the healthcare system; but these patients constitute a fragile, vulnerable and polymedicated group, where the prognostic evolution of VTE may vary ^10^.

Given that outpatient management has shown to have better outcomes compared to inpatient management, the dilemma seems to lie more in the effective management of comorbidities and/or intercurrent conditions of people, rather than focusing exclusively on the scope of the approach itself. In this sense, it is possible that VTE is a biological reserve marker, where the prognosis differs widely from RCT in a geriatric population. It is known that thrombosis is a clinical manifestation that precedes the diagnosis of other pathologies such as cancer. Real-world evidence is therefore valuable, because it reflects the diversity of the population and the complexities of healthcare in uncontrolled settings ^11^.

The presence of confounding by indication bias (defined as a systematic distortion in the results of a study due to differences in the baseline characteristics between the groups being compared ^12^, may arise when treatment allocation is not entirely random, but is influenced by factors related to the disease severity, comorbidities or other individual characteristics. As expected, among people with multiple concomitant comorbidities (e.g. cancer, obesity, COPD, CKD, HF) outpatient management was less common and nearly 50% of hospitalized patients had a moderate to severe Charlson score. Consistently, hospitalization was associated with more severe cases (explained by higher serological values of creatinine, BNP and troponin) as a proxy for baseline VTE risk stratification at diagnosis, which goes beyond the patient’s baseline clinical characteristics. Despite all these recently mentioned variables, hospitalization was independently associated with a significantly higher risk of mortality at 90 days compared to outpatient treatment (aHR: 1.99; 95% CI: 1.49-2.64; p=0.001).

Secondly, the 90-day mortality (25% overall, 43% in inpatients and 19% in outpatients) is strikingly high, and deserves special interpretation. While *a priori* it may seem to be an overestimated estimate (higher than expected by the research team), it should be clarified that this finding is consistent with other reports. On the one hand, a prospective cohort with data from 2006-2011 included 1736 cases in which pulmonary angiography, angiotomography or ventilation-perfusion scintigraphy were performed for a suspected diagnosis of PE, where only 504 were confirmed (prevalence of 29%) ^13^. In that study, the leading causes of death were PE in 60% of confirmed cases; while neoplasm (42%) and sepsis (37%) in the suspected group. However, the overall 90-day mortality was also high: 33% and 37%; respectively, suggesting that patients die equally during follow-up, regardless of the accurate diagnosis of ETV ^13^. Consistently, in a cohort between 2012-2014 that included 446 cases of VTE (with 292 adults aged 65 , representing 65%), the 90-day mortality rate in the elderly was 13% ^10^. Finally, a more recent study including 414 patients with a mean age of 61 years (minimum 18 and maximum 93), showed a mortality rate of 13.3% at 30 days and 21.8% at 90 days ^14^. On the other hand, our results also do not differ from other international studies that reported 18.9% 30-day mortality among patients ≥ 80 years ^15^; and 19.6% at one year ^16^.

It is noteworthy to mention that, out of a total of 2,293 deaths during 2017 in this same centre, only 32% were due to cancer, and regarding the place of occurrence, 80% occurred in an inpatient ward, suggesting that individuals and/or their families choose the hospital as the site for end-of-life care ^17^.

All this information underscores the increasing number of older adults with chronic conditions, which can limit end-of-life care options. As these conditions progress, patients and their families are faced with difficult decisions regarding acute intercurrences such as VTE. This highlights the importance of carefully considering the type of management and the patient’s background in shared clinical decision-making.

Thirdly, outpatient management was the most prevalent strategy (89% for DVT and 19% for PE), which aligns with findings from a US study reporting 57% and 18%, respectively ^3^. These results suggest that outpatient treatment has been more widely accepted for patients with DVT, while adoption for PE remains slower ^18^, possibly due to concerns about medication access, patient adherence, and uncertainty about outpatient follow-up (e.g., delays in accessibility).

It should be stressed that home hospitalization refers to the provision of healthcare services in the patient’s own home, rather than in a traditional hospital setting. However, although it shares certain characteristics with classical hospitalization (e.g.: the presence of healthcare staff), it resembles more to outpatient management (e.g. less close follow-up). This particular clinical scenario involves the provision of services by nursing staff during the first 48 hours, who deal with the subcutaneous application of enoxaparine, and patient education (so that they can then continue on their own) ^19^, which is complemented by monthly visits from a physician. In other words, in the management of VTE at our institution home hospitalization serves as an administrative measure to ensure bed availability and medication coverage (provided 100% free of charge), without introducing bias related to differential clinical follow-up. In recent years, this option has become increasingly common due to the rising demand for acute care beds ^20^, but undoubtedly used more frequently for DVT than for EP ^21^. Some studies have even assessed patient satisfaction and the cost-effectiveness of this alternative ^22^.

It cannot be overlooked that only 25 patients received DOACs ^23^, which allows us to reflect once again on the discrepancies between RCTs that are conducted under highly controlled conditions and select participants with specific and restrictive criteria, but which undoubtedly contrast with real-world evidence where more representative situations from daily clinical practice are obtained. For example, the apixaban trial for PE included 2691 with an mean age of 57 years and 2.5% active cancer ^24^, while for DVT it included 1731 with a mean age of 55 years and 6.8% active cancer ^25^. Similarly, the dabigatran trial for VTE included 1273 subjects with a median age of 56 and 5% cancer history ^26^. This raises questions: is there underuse of these drugs due to clinical prescribing inertia (tendency of professionals to maintain unchanged treatment)?; is there a lack of updating in professional training? or, simply, do the RCT results apply to our healthcare population? ^27^.

Regarding the 15% initially managed on an outpatient basis who were later hospitalized, this figure coincides with the 13% reported in a closed retrospective cohort during 2014-2015 ^28^. These indicators play a crucial role in healthcare ^29^, and the key lies in determining the avoidability of readmission or prioritizing opportunities to improve quality of care ^30^^,^^31^.

Regarding recurrence and bleeding, there were no significant differences between the groups, and the overall rates were low (0.67% and 2.83%, respectively), consistent with other reports mentioning occurrences below 2%, reinforcing evidence on the safety of outpatient management ^5^.

The main strength of this study is the contribution of local real-world evidence with a large number of cases of VTE in a contemporary setting ^32^. It cannot be overlooked that the ICD-10 code search was complemented with expert validation of the confirmed case, and some variables (e.g., initiated treatment) were collected through manual review rather than automatic capture (due to underreporting). Finally, statistical techniques such as adjustment for confounding variables were applied, allowing for more precise estimates.

However, it has several limitations inherent to the design itself and the handling of retrospective data, such as indication bias, which occurs when treatment assignment is influenced by the presence of certain health conditions or participant characteristics. Additionally, being single-center and restricted to institutional affiliates may lead to selection and information bias, and it compromises external validity and data extrapolation (e.g., racial/ethnic differences for other countries, and/or socio-economic level even for our own country).

In conclusion, in a cohort of elderly patients with VTE and high burden of comorbidities, outpatient management was shown to have fewer adverse events than inpatient management. Thus, the dilemma would not seem to be in the management setting per se, but in the management of disease burden, where it is not only possible that VTE is a marker of biological reserve, but where evolution or prognosis differs radically from RCTs.
